# Aspiration techniques for bronchoalveolar lavage in translational respiratory research: Paving the way to develop novel therapeutic moieties

**DOI:** 10.14440/jbm.2017.174

**Published:** 2017-07-03

**Authors:** Kamal Dua, Shakti D. Shukla, Philip M. Hansbro

**Affiliations:** 1 Discipline of Pharmacy, Graduate School of Health, University of Technology Sydney, Sydney 2007, NSW, Australia; 2 Priority Research Centre for Healthy Lungs, Hunter Medical Research Institute, Lot 1 Kookaburra Circuit, New Lambton Heights, Newcastle, NSW 2305, Australia; 3 School of Biomedical Sciences and Pharmacy, The University of Newcastle, Callaghan, NSW 2308, Australia

**Keywords:** bronchoalveolar lavage, aspiration, respiratory

## Abstract

Bronchoalveolar lavage (BAL) is a simple, yet informative tool in understanding the immunopathology of various lung diseases *via* quantifying various inflammatory cells, cytokines and growth factors. At present, this traditional method is often blended with several robust and sophisticated molecular and biological techniques sustaining the significance and longevity of this technique. Crucially, the existence of slightly distinct approaches and variables employed at different laboratories around the globe in performing BAL aspiration indeed demands an utmost need to optimize and develop an effective, cost-effective and a reproducible technique. This mini review will be of importance to the biological translational scientist, particularly respiratory researchers in understanding the fundamentals and approaches to apply and consider with BAL aspiration techniques. This will ensure generating a meaningful and clinically relevant data which in turn accelerate the development of new and effective therapeutic moieties for major respiratory conditions.

## INTRODUCTION

Globally, chronic respiratory diseases such as asthma, chronic obstructive pulmonary disease (COPD) and cystic fibrosis are among the leading causes of mortality and morbidThe main rationale and objective for collecting the BALf from widely used mice ity. Currently available treatments for these diseases only focus on alleviating various symptoms instead of completely preventing/treating the diseases, which warrants ongoing investigations for novel targeted treatments [1]. To establish an effective therapeutic moiety for various lung diseases, it is crucial to have a thorough knowledge on various cellular and immunological processes and parameters in lungs [2,3]. One such approach includes sampling bronchoalveolar lavage fluid (BALf), which aids in the assessment of immune cells, as well as various cytokines and mediators produced by these immune cells and the respiratory epithelial cells.

With bronchoalveolar lavage (BAL) method, various cellular and non-cellular components from the respiratory tract (epithelial lining fluid) can be recovered [4]. Despite being a traditional approach, it avoids lung biopsies in various human diseases like pulmonary proteinosis, alveolar haemorrhage and eosinophilic pneumonia. Apart from these, the application of BAL includes diagnosis of various infectious diseases such as ventilator-associated pneumonia, mycobacterial infection, aspergillus fumigatus infection, pneumocystis pneumonia, chlamydia pneumoniae pneumonia, cryptococcal infection and non-infectious diseases like idiopathic lung fibrosis, connective tissue disorders, alveolar haemorrhage, alveolar proteinosis, *etc*.

The historical concept of BAL started in early 1920s, when it was found particularly useful in the treatment of phosgene gas poisoning [5]. At present, evaluation of BAL remains one of the most commonly employed techniques in both diagnosis and research in respiratory disorders to gain meaningful cytological information [6,7]. The major advantage of collecting BALf is minimal invasion of patient, which is the most important basis of human and veterinary medicine [8].

Despite the immense clinical utility of BAL in the evaluation of respiratory diseases, our understanding is limited to establishing and optimizing a simple, effective, affordable and reliable BAL aspiration method at laboratory scale. Thus, it is of utmost importance to review the currently employed common practices of BAL acquisition in laboratory animals and how this useful technique enables the in-depth understanding of respiratory diseases and research. This mini review primarily focuses on the importance and approaches employed in various published studies for BAL collection and will benefit various biological translational and medical researchers, particularly in the field of respiratory diseases.

## CLINICAL UTILITY OF BALf COLLECTION

The BALf collection in human patients involve inserting a flexible tube connected to a mini-camera (commonly known as fiberoptic bronchoscope) into the trachea to enable real-time visualization and operating the instrument at the appropriate site, post administration of local anesthesia [9]. Rosell *et al*. also recommended using plastic tubing between the 50 ml syringe and the working channel of the flexible bronchoscope when carrying out manual instillation and suction [10]. In most cases, three to five aliquots of 50 ml of sterile, phosphate-buffered saline solution at 37°C is instilled and after each instillation, the fluid is gently aspirated with a negative pressure of **−**40 to **−**50 mm Hg [11]. Recently, a systemic review of published literature upto 2016 concluded that the process of collecting BALf *via* flexible bronchoscope in humans is generally safe, however, it also asserted the need of more research to document complications of procedure in greater details [12].

The main rationale and objective for collecting the BALf from widely used mice models of respiratory diseases is to evaluate differential cell counts, which is clinically significant in understanding and underpinning various process and mechanisms involved in inflammation, primarily at cellular level, but also in elucidating disease pathology at large [13-17]. This is an approach which also helps in obtaining a correlation between the BAL differential cell counts and various airway responsiveness parameters such as airway resistance, diffusing capacity of the lungs for carbon monoxide (DLCO) *etc.*, which ultimately predicts the pathophysiological consequences occurring during various stages of the chronic inflammatory airway diseases.

Several attempts have been made to critically evaluate various aspiration techniques to optimize and have a reliable approach for the collection of BALf in various animal models of airway diseases. One such study by Woods *et al*. [8] involved collection the BALf using manual aspiration (MA) and suction pump aspiration (SPA) in dogs with pulmonary disease, and then comparing the sample quality of the collected BALf. They have shown that SPA aids in better BALf retrieval than MA, however, it does not affect the diagnostic parameters. Similarly, Hoffman have investigated various sampling techniques and developed guidelines for cytologic preparation and analysis in equine airways [18].

Many researchers have postulated various methods to carry out intubation of the mouse lung in order to assess both pulmonary function and BAL in the individual mice with longitudinal studies [19-23]. Such techniques appear to be largely successful but they are quite expensive and require intense training for the operator. Notably, Das *et al*. reported a simplified approach for intubating mice in longitudinal studies where the study involved limited number of expensive research animals [24]. They demonstrated usage of fiber-optic light source to visualize trachea which makes the technique convenient to the researcher and aids in measurement of lung mechanisms for relatively longer duration (approximately several weeks) [24]. Crucially, this method could facilitate repeated BALf collection in rats that could reveal real time cellular/cytokine profile changes in the models of chronic respiratory diseases [25].

Maxeiner and co-workers standardized a method in murine model of lung carcinoma and melanoma which assists in understanding and analyzing various cells and released mediators in natural state [26]. Analyzing BALf is one of the prime parameters to understand the underlying disease pathology in prominent mice models of major respiratory diseases, including asthma [27-29], COPD [30,31], and pulmonary fibrosis [32]. Moreover, the application of BALF is not only restricted to quantifying the inflammatory cells, it is also employed to determine the viral titre in various viral infections [3,30].

## VARIABLES AFFECTING BALf COLLECTION

The most common problem encountered by researchers during the BALf collection is the variability in the sampling volume [33], which, in turn, significantly affects the amount of cells and fluid returned. It has also been reported that various sections of the lungs have dissimilar BALf findings [34,35]. There are various factors which may explain this variability (**Table 1**) such as: the underlying disease [36], amount and the nature of instillation used [37], size of syringes [38,39], amount of “dwell” time of instillation before aspiration [38-41], extent of pressure used while carrying out the BALf collection [42], number of aliquots and handling of the collected BALf. Importantly, there are set of recommendations been laid down by The European Respiratory Society (ERS) which clearly explains the major causes of the variability and the strategies to overcome these variabilities [43], as well as various methods to perform BAL [44,45].

The various solution and media generally employed for collecting BALf include normal sterile saline (0.9% NaCl) [46]/phosphate buffer saline (PBS) [47,48] and Hanks buffered salt solution (HBSS) [49]. Also, the volume and number of aliquots employed for BAlf varies from one study to another, ranging from 500 µl [47] to 1000 µl [49]. All these parameters need to be thoroughly considered based on the previously reported studies to ensure the reproducibility and accuracy of the BALf technique.

Song *et al*. standardized BAL method based on the suction frequency number and lavage fraction number in rats [50]. Similarly, Singletary *et al*. [41] compared two different aspiration techniques: conventional method which involves the use of syringe by manually installing and aspirating the fluid and modified method where insertion of a sterile intravenous extension tubing was done between the syringe tip and bronchoscope biopsy channel. Among both the methods, the modified method was found to be statistically significant in the amount of instillation collected and cells recovered (8.3% increase) as compared to the conventional method, which proved to be highly reproducible and of clinical importance in understanding the disease pathology [41].

## RECENT DEVELOPMENTS IN BALf ANALYSIS

The recent advances in the BALf analysis include the proteomic analysis of various proteins which have provided information on the mechanism involved with ultrafine carbon black-induced lung injury in mice [51]. Remarkably, considerable progress has been made in the development of a validated computer program based on polychotomous logistic regression model, which is a validated model and have better diagnostic power of BALf analysis and reliable prediction of the current diagnosis in patients with interstitial lung diseases [52,53]. Such models can also be efficiently employed in various translational studies where the set of reliable and reproducible BALf data can be obtained.

Radhakrishnan *et al*. briefed a translational research on BALf which have provided a detailed information on the identification of clinically relevant microbiologic pathogen and cellular analysis in pediatric respiratory diseases [54]. Also, a candidate molecular biomarkers in idiopathic pulmonary fibrosis (IPF) namely, S100A9 protein is been identified in the BALf proteins [55]. Additionally, Correlating Outcomes With Biochemical Markers to Estimate Time to Progression in Idiopathic Pulmonary Fibrosis (COMET) study [56-58] analysis with BAlf have shown an association in the development of IPF and presence of Staphylococcus and Streptococcus genera [57] in the United States. Furthermore, Molyneaux and co-workers have shown increased pathogenic load of *Haemophilus*, *Streptococcus*, *Neisseria* and *Veillonella spp*. in IPF patients as compared to control groups (healthy smokers, non-smokers, and patients with moderate COPD) [59].

Ortea *et al*. had used SWATH MS data-independent acquisition and targeted data extraction where they have discovered a protein biomarker in BALf which can be used in the diagnosis, prognosis and subtyping of lung cancer including the treatment response monitoring [60]. All such listed BAlf analysis studies have provided a great platform and foundation for understanding various other respiratory diseases so as to identify the mechanisms involved and new therapeutic interventions.

## CONCLUSION AND FUTURE PROSPECTS

BAL is an extremely important respiratory clinical sample and is useful in extracting a great level of information on the disease markers by blending with various modern and sophisticated molecular and biological techniques like –omics approach such as proteomic and transcriptomics analysis (aids in establishing the detailed mapping of the cellular output and gene displays in the BAL fluid and cells in both naïve and patient group) [61,62], enzyme-linked immunosorbent assay (ELISA) [63], flow cytometric analysis [64], immunohistochemistry [65], quantitative polymerase chain reaction, (qPCR) [66] mass spectrophotometry [67,68] and nuclear magnetic resonance (NMR) [68] (**Table 2**). Optimizing BALf collection and combining it with modern advanced techniques would ensure higher sensitivity in diagnosing and evaluating a variety of inflammatory processes in lungs by quantifying the different inflammatory cells, assessing various inflammatory cytokines and growth factor expression to underpin the mechanism involved, disease features and pathology.

Remarkably, various special cell types such as dendritic cells could also be specifically isolated from BALf which would further our understanding on specific innate immune pathways [69-71].

In order to have a clinically relevant and meaningful data, it is essential to validate and standardize the lavage process and subsequent handling/processing to warrant greater reproducibility and minimum variability for future applications in respiratory research. The recent advances in technology, various computer assisted programs also have enhanced reproducibility and accuracy in the BALf aspiration techniques.

## Figures and Tables

**Table 1.** Various factors causing variability with bronchoalveolar lavage sampling.

Serial No.	Factor causing variability with BAL sampling
1	The underlying disease
2	Extent of pressure applied while collecting BALf
3	Amount and nature of instillation used
4	Number of aliquots
5	Size of syringes used
6	Amount of “dwell” time of instillation before aspiration
7	Handling and storage of collected BALf

**Table 2.** Advances in analysis of bronchoalveolar lavage.

	BAL aspiration
Conventional approaches	Modern approaches
Cytology	Proteomic analysis
	Enzyme-linked immunosorbent assay
	Flow cytometric analysis
	Immunohistochemistry
	Quantitative polymerase chain reaction
	Mass spectrophotometry
	Nuclear magnetic resonance
